# Hypolipidemic and Hepatoprotective Effects of High-Polydextrose Snack Food on Swiss Albino Mice

**DOI:** 10.1155/2020/5104231

**Published:** 2020-03-14

**Authors:** Jin Han Yang, Thi Thu Tra Tran, Van Viet Man Le

**Affiliations:** Department of Food Technology, Ho Chi Minh City University of Technology Vietnam National University-Ho Chi Minh City (VNU-HCM) 268 Ly Thuong Kiet, District 10, Ho Chi Minh, Vietnam

## Abstract

In this study, the hepatoprotective and hypolipidemic effects of high-polydextrose snack food on Swiss albino mice were investigated. The mice were randomly divided into three groups: control diet, high-fat diet, and high-fat and fiber diet groups. Addition of high-polydextrose snack to the high-fat diet resulted in significant reduction in the liver weight, the accumulation of lipid droplets in liver, and the liver damage of hyperlipidemic mice in comparison with the high-fat diet. The high-polydextrose snack also decreased the content of total triglyceride, cholesterol, and low-density lipoprotein cholesterol as well as the alanine aminotransferase and aspartate aminotransferase activities in the mice serum. In addition, the high-polydextrose snack significantly increased the high-density lipoprotein cholesterol content of the hyperlipidemic mice. Consequently, use of high-polydextrose snack generated hepatoprotective and hypolipidemic effects on hyperlipidemic mice.

## 1. Introduction

Public awareness on health and food safety has been increased, and the concept of a fiber-rich diet is gaining great importance due to digestive health benefits [1]. Dietary fiber deficiencies in human nutrition are related with several diseases such as colon cancers, constipation, cardiovascular diseases, and diverticulosis [2]. Dietary fiber could reduce energy intake, increase immune system, improve intestinal microbiota activity, and enhance postprandial lipid responses [3]. Among different commercial dietary fiber preparations used in food industry, polydextrose is a soluble functional fiber with prebiotic activity. Polydextrose is a polymer of glucose with the degree of polymerization from 2 to 120 and *α* and *β* (1–2), (1–3), (1–4), and (1–6) glycosidic bonds [4]. It is reported that polydextrose improves the growth of some intestinal bacteria such as *Clostridium difficile* and *Bifidobacterium* which provide physiological benefits to human health [4]. The impacts of polydextrose digestion on postprandial insulin and glucose response were investigated in numerous studies. With a low glycemic index (4–7), polydextrose is proved to decrease the potential of blood glucose level rising in the human body [5]. Polydextrose can be used to replace sugar for low-glycemic diet [1]. In addition, polydextrose intake reduces glucose absorption from the small intestine and improves bowel function for health [6]. Moreover, it also decreases the total and low-density lipoprotein cholesterol levels in blood serum.

These physiological and functional benefits result in great interest to apply polydextrose to the development of healthy food products [1]. Among food products, snacks are worldwide popular for both children and adults. Nevertheless, the dietary fiber content in extruded snack food conventionally varied from 4.0 to 5.2% and the snack food is considered as low-fiber food [7]. Various studies have been performed to increase the fiber content of extruded snack food, whereas it is not well known how snack fibers affect health benefit through animal testing. Particularly, the effects of extruded snack food enriched with polydextrose on hyperlipidemia and hepatoprotection on pathological model mice have not been stated yet. In this study, the hyperlipidemia mice model was selected with a high-fiber diet to investigate the possible hypolipidemic and hepatoprotective effects of snack food enriched in polydextrose on hyperlipidemic mice.

## 2. Methods and Materials

### 2.1. Materials

High-fiber snack for mice feeding was produced from 70% corn meal and 30% polydextrose (the ratio was calculated on dry weight basis). The experiments were performed using a twin-screw extruder (MPF 80/15 model, APV Baker, Peterborough, United Kingdom) with a productivity of 300 kg/h [7]. The extruded products were immediately sealed for analysis and mice feeding. The high-fiber snack contained 53% carbohydrate, 30% total fiber, 4% protein, 0.4% lipid, and 0.6% ash.

Anifood supplied by Nha Trang Pasteur Institute (Khanh Hoa province, Vietnam) was used as the basic diet. Anifood contained 58–63% carbohydrate, 5–6% total fiber, 21–23% protein, 5–7 lipid, and 6–8% ash. Lard (45.1% of monounsaturated fat, 11.2% of polyunsaturated fat, and 39.2% of saturated fat) was originated from Saigon Coop Group (Ho Chi Minh City, Vietnam). Yolk powder (44% protein and 35% lipid) was provided by Golden Egg Ltd. (Ho Chi Minh City, Vietnam). Cholesterol (92.5% purity) and sodium cholate acid (98% purity) were supplied by Sigma-Aldrich Ltd. (Missouri, USA).

### 2.2. Experimental Design

Fifteen male Swiss albino mice (31.2 ± 0.41 g) were used for the experiment. They were provided by the Ho Chi Minh City Pasteur Institute (Ho Chi Minh City, Vietnam). The mice were kept under the average husbandry environment with a 12 h light-dark cycle (8 : 00 to 20 : 00) for two weeks to acclimate with the laboratory environment. They were supplied ad libitum with a basic diet and distilled water (feeding time at 9 : 00–10 : 00 everyday). Then the mice were casually divided into three groups (five mice per cluster).

Group 1 (control diet): the mice were nourished with a basic diet.

Group 2 (high-fat diet): the mice were served with a high-fat diet.

Group 3 (high-fat and fiber diet): the mice were served with a high-fat and fiber diet.

The mixing formula for mice feeding and approximate composition of each diet group are presented in Tables 1 and 2, respectively.

The experimental procedure was strict compliance with the Declaration of Helsinki (1964). During the test period, the amount of feed intake and the body weight of each mouse were recorded weekly. At the end of the twelfth week, all mice were anesthetized with diethyl ether and then euthanized by carbon dioxide. Their blood was collected from their heart for biochemical analysis, while the liver was detached from each corpse for weight measurement and pathological examination.

### 2.3. Serum Biochemical Parameters

At the end of the experimental intervention, mice were euthanized under anesthesia after 12 h fasting. Blood was collected into heparinized tubes, and the plasma was then separated by centrifugation (Centrifuge 5417R, Eppendorf, Germany); the centrifugation speed and time were 3,000 rpm and 10 min, respectively. Lipid profile including total cholesterol, triglycerides, high-density lipoprotein cholesterol, low-density lipoprotein cholesterol, and plasma levels of hepatic enzymes such as aspartate transaminase and alanine aminotransferase were determined by using commercial diagnostic kits (ZRT, Oregon, United States) according to the manufacturer's instruction. An Accu-Chek Performa (Roche Diagnostics, Mannheim, Germany) test strip was used to measure the blood glucose level.

### 2.4. Histopathological Studies of Liver

The liver was separated, washed delicately in 9 g/L NaCl solution, blotched with the paper funnel, gauged, and solidified at−70°C [8]. Histological test for the liver was performed with hematoxylin and eosin staining. The samples were preserved in 10% formalin for 6 hours and dried out before embedding in paraffin wax; they were then sectioned at 4–6 *μ*m thick for staining with hematoxylin and eosin [9]. The liver sections were then observed under a microscope by human eyes for the estimation of extent of hepatic damage.

### 2.5. Statistical Analysis

Statistical analysis was conducted using Statgraphics Centurion XV (Statpoint Technologies Inc., Warrenton, Virginia, USA). The experimental data were expressed as mean ± standard deviation. The difference between the means of different groups was determined using Tukey's comparison test with a significance level set at *p* < 0.05.

## 3. Results and Discussion

### 3.1. Feed and Energy Intake

**Figure 1:**
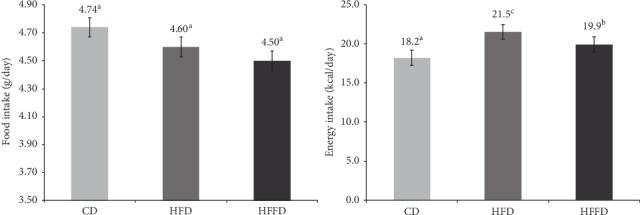
Daily feed and energy intake of Swiss albino mice during the twelve-week experiment (CD: control diet group, HFD: high-fat diet group, and HFFD: high-fat and fiber diet group). The data are mean value ± standard deviation (*n* = 5). Values with different small letters in the figure are significantly different (*p* < 0.05).

Daily feed and energy intake of each mouse of the three mice test groups are shown in Figure 1. The statistical analysis showed that the average feed intake among the three groups was similar (Figure 1(a)). However, the energy intake was significantly different among the three clusters (Figure 1(b)). The daily energy intake of the high-fat diet group was somewhat higher than that of the high-fat and fiber diet group, while this value of the control diet group was the lowest. Similar observation was reported by Kumar et al. [10]; the inulin oligofructose-diet attenuates energy intake in high-carbohydrate and high-fat diet-fed rats.

### 3.2. Effects of High-Polydextrose Snack on Body Weight

The average body weight of mice during the experiment is shown in Table 3. At the beginning of the experiment, the three mice groups showed insignificant difference in body weight. Since then, the body weight of all mice gradually increased during the twelve weeks. However, from the second week, the body mass of the high-fat diet group was significantly greater than that of the control diet and the high-fat and fiber diet group. At the end of the twelfth week, the mice fed with the high-fat diet showed a significantly higher weight gain than those with the control diet. When snack with high polydextrose content was added to the high-fat diet, the extra weight gain generated by the high-fat diet was eased (*p* < 0.05). The final body weight of polydextrose diet mice was slightly higher than that of the control diet group. The weight of the high-fat diet mice was roughly 12.9% and 6.7% higher than that of the control diet and high-fat and fiber diet mice, respectively. Similarly, recent studies demonstrate that wheat fiber and phytochemicals isolated from wheat bran suppress body weight gain in high-fat diet-fed mice [11]. In addition, bamboo shoot fiber also shows an effective role in suppressing high-fat diet-induced obesity than soluble fibers [12]. On the contrary, Weitkunat et al. [13] reported that dietary fibers are discussed as factors influencing diet-induced obesity and energy homeostasis. Paradoxically, there are some evidence on both prevention and promotion of obesity development. It could be assumed that the provision of additional energy by fermentation of the dietary fiber is a possible mechanism contributing to the increase in body weight.

### 3.3. Effects of High-Polydextrose Snack on the Lipid Profile and Serum Parameters of the Mice

The lipid profile and glucose concentration of mice serum are presented in Table 4. The serum triglyceride content of mice in the high-fat diet group was 2.6 and 2.0 times higher than that in the control diet and the high-fat and fiber diet group, respectively. Similar observation is reported when the chocolate containing polydextrose is fed to mice. The mice show reduced serum triglyceride levels, which would indicate that polydextrose abridged either the level of fat preoccupation in the earlier portion of the small intestine or indorsed big transfer time via the gut [14]. In addition, Albarracín et al. [15] report that the triglycerol content in mice liver is decreased by 17% when polydextrose is added to the diet during a 60-day experiment.

Total cholesterol and low-density lipoprotein cholesterol contents are possibly associated with coronary heart disease [16]. Low-density lipoprotein cholesterol is referred as bad cholesterol since it can deliver fat molecules to artery walls, which attracts macrophages and thus drives atherosclerosis. Likewise, high-fat diet is demonstrated to increase the total cholesterol and low-density lipoprotein cholesterol content in the body [17]. As expected, the total cholesterol and low-density lipoprotein cholesterol concentrations in the serum of the high-fat diet group were 1.3 and 2.0 times, respectively, higher than those of the high-fat and fiber diet group (Table 4). Similar trend of decrease in total cholesterol and low-density lipoprotein cholesterol content is observed when healthy adults are administered with 10 g polydextrose per day during an 18-day experiment [18]. In addition, the high-density lipoprotein cholesterol content in the serum of high-fat diet group was meaningfully lower than that of the control diet and the high-fat and fiber diet group. As a consequence, the high-fat and fiber diet reduced hyperlipidemia in mice.

Table 4 also shows that the glucose level of the high-fat diet group and the high-fat and fiber diet group was 2.6 and 1.6 times, respectively, higher than that of the control diet group. The obtained results indicate that polydextrose would definitely have a positive role in reduction in postprandial glucose absorption. A similar phenomenon is reported in human test subjects; according to Jie et al. [6], the diet with 50 g glucose results in a glycemic index of 100% while that with 50 g glucose and 12 g polydextrose generates the glycemic index of 89%.

### 3.4. Effects of High-Polydextrose Snack on Liver Function

The measurement of alanine aminotransferase and aspartate aminotransferase activities in serum can directly indicate the level of hepatic damage as well as the progression of fatty liver induced by high-cholesterol diet [19].  Table 5 shows alanine aminotransferase and aspartate aminotransferase activities in the mice serum. The aspartate aminotransferase and alanine aminotransferase levels of mice treated with high-fat diet were 79% and 83%, respectively, higher than those of mice with the control diet. Nevertheless, the mice with the high-fat and fiber diet and the control diet had statistical similarity in alanine aminotransferase and aspartate aminotransferase activities. It is reported that nonstarch polysaccharides have a lowering effect on the alanine aminotransferase and aspartate aminotransferase activities in mice serum since they have hypoglycemic and hypolipidemic effects on intestinal digestion and nutrient absorption for diabetic rats [20]. It can be inferred that polydextrose has hepatoprotective effect on high-fat diet-fed mice.

The three diet groups showed significantly different indexes of the liver (*p* < 0.05) as given in Table 5. The liver weight of the high-fat diet-fed mice was 51% and 36% higher than that of the control diet-fed and high-fat and fiber fed mice, respectively. In addition, fat mass of the high-fat diet-fed mice was 138% and 37% higher than that of the control diet and high-fat and fiber diet-fed mice, respectively. This result indicates that dietary-fiber diet reduces the risk of hepatic steatosis in mice. In addition, dietary fiber also causes metabolic effects by decreasing fat storage because of increased satiety [9]. Similar results are reported when bamboo shoot fiber is used in mice test [12].

### 3.5. Effects of High-Polydextrose Snack on Fat Accumulation and Liver Structure

**Figure 2:**
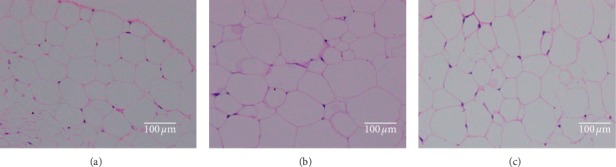
Histological analysis of epididymal fat tissue of three groups. Epididymal adipose segment; the tissues were magnified by 200 times: (a) control diet group; (b) high-fat diet group; (c) high-fat and fiber diet group.

Histological analysis of epididymal fat tissue of the three groups is shown in Figure 2. At the end of the twelve-week experiment, epididymal fat accumulation of the high-fat diet group was higher than that of the control diet group. Supplementation with the high-fat and fiber diet obviously showed smaller adipocyte size in the liver tissue. Similar results are reported when insoluble dietary fiber from pear pomace is used in high-fat diet for rats [17].

**Figure 3:**
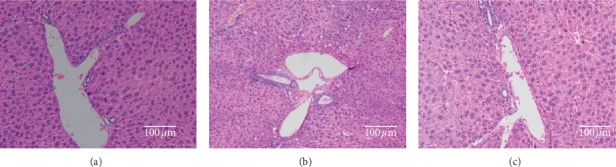
Micrographs of liver tissue of mice for 3 groups (×100): (a) control diet group; (b) high-fat diet group; (c) high-fat diet and fiber diet group.

Hematoxylin and eosin stained paraffin sections indicated normal hepatic architecture in clear hepatic sinusoid and clear hepatic lobule in the livers of the control diet-fed mice (Figure 3(a)), whereas pathological symptoms were indicated in the high-fat diet-fed mice. With large and abundant lipid droplets in parenchymal cells, the liver cells appeared to have serious fatty degeneration along with inflammatory cell infiltration (Figure 3(b)). When the mice were fed with snack enriched in polydextrose for twelve weeks, the liver cell degeneration was ameliorated (Figure 3(c)). Consequently, polydextrose had a positive effect on hepatic fatty degeneration in high-fat diet mice. Similarly, supplementation of resistant maltodextrin effectively reduces the histopathological changes in the liver of high-fat diet-fed rats [21]. Nevertheless, Janssen et al. [22] reported that feeding of guar gum for 18 weeks protected mice from diet-induced obesity but eventuated hepatic inflammation and disrupted enterohepatic circulation. Therefore, further studies on different fiber types are essential to clarify their impacts on histopathological changes in the liver of high-fat diet-fed mice.

## 4. Conclusion

The findings in the present study indicated that the use of snack food enriched in polydextrose resulted in significant reduction in the body weight of mice induced by the high-fat diet. The high-fat and fiber diet also decreased the liver weight, the accumulation of lipid droplets in liver, and the liver damage of the hyperlipidemic mice. On the other hand, the use of high-polydextrose snack in the high-fat diet reduced the content of triglyceride, total cholesterol, and low-density lipoprotein cholesterol as well as the alanine aminotransferase and aspartate aminotransferase activities in the mice serum. Moreover, the diet with polydextrose added snack increased the high-density lipoprotein cholesterol content in the mice serum. Accordingly, the diet with polydextrose added snack generated the hepatoprotective and hypolipidemic effects on hyperlipidemic mice.

## Figures and Tables

**Table 1:** Approximate composition (calculated on % dry weight basis) of each diet group.

Compounds	Control diet	High-fat diet	High-fat and fiber diet
Carbohydrate	58–63	46–50	46.5–50.7
Protein	≥21	≥22	≥19.3
Fat	5–7	18.5–21.1	15.8–18.0
Fiber	5–6	4.0–4.8	7.9–8.6
Mineral	6–8	5.6–6.4	4.9–5.6

**Table 2:** The mixing formula for mice feeding.

Control diet (CD)		High-fat diet (HFD)		High-fat and fiber diet (HFFD)	
Ingredient	Ratio (%, w/w)	Ingredient	Ratio (%, w/w)	Ingredient	Ratio (%, w/w)
Basic diet	100	Basic diet	80	Basic diet	65
		Lard	12.5	Lard	12.5
		Yolk powder	5	Yolk powder	5
		Cholesterol	2	Cholesterol	2
		Sodium cholate acid	0.5	Sodium cholate acid	0.5
				Snack form cornmeal and polydextrose	15
Energy intake (kcal/kg)	3,840		4,627		4,410

**Table 3:** Change in body weight of mice (*g*) during the twelve-week experiment.

Time (weeks)	Control diet group	High-fat diet group	High-fat and fiber diet group
0 (^*∗*^)	30.9 ± 0.51^aA^	31.4 ± 0.41^aA^	31.2 ± 0.30^aA^
2	31.4 ± 0.55^abA^	32.7 ± 0.53^bA^	32.0 ± 0.31^bB^
4	31.8 ± 0.55^bcA^	33.8 ± 0.52^cB^	32.7 ± 0.28^cC^
6	32.3 ± 0.51^cdA^	35.0 ± 0.66^dB^	33.6 ± 0.36^dC^
8	32.9 ± 0.55^deA^	36.2 ± 0.60^eB^	34.6 ± 0.34^eC^
10	33.5 ± 0.48^efA^	37.4 ± 0.79^fB^	35.3 ± 0.37^fC^
12	33.9 ± 0.52^fA^	38.3 ± 0.64^gB^	35.9 ± 0.35^gC^

The data are mean value ± standard deviation (*n* = 5). Values with different lowercase letters in the same column are significantly different (*p* < 0.05). Values with different uppercase letters in the same row are significantly different (*p* < 0.05), and 0 (^*∗*^) means the initial moment of the experiment.

**Table 4:** Serum lipid profile and glucose concentration of mice in the three diet groups.

Mice group	Triglyceride (mg/dL)	Total cholesterol (mg/dL)	HDL cholesterol (mg/dL)	LDL cholesterol (mg/dL)	Glucose (mg/dL)
CD	129.9 ± 21.9^a^	148.9 ± 10.1^a^	82.1 ± 8.6^b^	41.5 ± 5.7^a^	59.8 ± 13.2^a^
HFD	334.6 ± 32.4^b^	224.8 ± 10.8^c^	57.0 ± 6.2^a^	101.5 ± 8.7^c^	158.4 ± 21.6^c^
HFFD	164.2 ± 13.2^c^	177.8 ± 9.7^b^	94.4 ± 9.5^c^	51.4 ± 3.2^b^	97.4 ± 8.9^b^

The data are mean value ± standard deviation (*n* = 5). Values with different small letters in the same column are significantly different (*p* < 0.05). CD: control diet group, HFD: high-fat diet group, and HFFD: high-fat and fiber diet group. HDL and LDL cholesterol is high-density lipoprotein and low-density lipoprotein cholesterol, respectively.

**Table 5:** Liver weight, fat mass, AST, and ALT of the mice.

Mice group	Liver weight (g)	Fat mass (g)	AST (U/L)	ALT (U/L)
CD	1.44 ± 0.05^a^	0.42 ± 0.09^a^	132.1 ± 28.3^a^	64.7 ± 9.3^a^
HFD	2.17 ± 0.14^c^	1.00 ± 0.18^c^	236.6 ± 15.5^b^	118.5 ± 22.7^b^
HFFD	1.59 ± 0.06^b^	0.73 ± 0.08^b^	144.3 ± 25.9^a^	59.3 ± 12.7^a^

The data are the mean values ± standard deviation (*n* = 5). Values with different small letters in the same column are significantly different (*p* < 0.05). CD: control diet group, HFD: high-fat diet group, HFFD: high-fat and fiber diet group, AST: aspartate aminotransferase, and ALT: alanine aminotransferase.

## Data Availability

The data used to support the findings of this study are available from the corresponding author upon request.
